# Gastrocolonic fistula: a rare complication of upper gastrointestinal surgery

**DOI:** 10.1093/jscr/rjab181

**Published:** 2021-05-14

**Authors:** Trudy Hong, Dion Koh, Andrew Gray

**Affiliations:** Department of General Surgery, Monash Health, Clayton, Victoria, Australia

## Abstract

A gastrocolonic fistula is a rare surgical presentation, typically in the setting of gastric or colonic malignancy. This report presents the first reported Australian case of a gastrocolonic fistula following upper gastrointestinal surgery.

A middle-aged woman presented to emergency with a short history of severe abdominal pain, faeculent vomiting, profuse diarrhoea and weight loss. This was in the setting of a previous pylorus-preserving pancreaticoduodenectomy complicated by marginal ulceration, for which a distal gastrectomy and Roux-en-Y reconstruction was required.

The rarity of gastrocolonic fistulae and non-specific presentation with diarrhoea, vomiting and weight loss can make the diagnosis challenging. The mainstay of management is surgical resection in both benign and malignant disease.

## INTRODUCTION

A gastrocolonic fistula is a pathological communication between the stomach and the colon. It is most commonly seen in the setting of gastric or colonic malignancy, and in exceedingly rare cases, gastrocolonic fistulae can also develop as a complication following upper gastrointestinal surgery. Here we present the first Australian case of a gastrocolonic fistula following upper gastrointestinal surgery.

## CASE REPORT

A 48-year-old lady presented to the emergency department (ED) of a metropolitan tertiary hospital with a short history of severe abdominal pain, faeculent vomiting, profuse diarrhoea and weight loss. This was on the background of a complex surgical history characterized by a pylorus-preserving pancreaticoduodenectomy 6 years beforehand for a gastrinoma at the pancreatic head. This was complicated by recurrent ulceration at the duodenojejunostomy anastomosis for which the patient underwent a distal gastrectomy and Roux-en-Y reconstruction 1 year prior. Despite this aggressive surgical management, repeat endoscopy 6 months prior demonstrated persistent marginal ulceration at the gastrojejunostomy as well as development of a further jejunal ulcer despite strict adherence to a twice-daily regimen of high-dose proton-pump inhibitor as well as the addition of sucralfate. As such, the patient was planned for a total gastrectomy however this was delayed due to the COVID-19 pandemic and, as it would be, she presented to ED before this elective surgery could be performed. Her other past history was significant for Crohn’s disease, gastroesophageal reflux, Raynaud’s disease and a previous right hemithyroidectomy for a neuroendocrine tumour.

On presentation to ED, the patient appeared unwell and was tachycardic with a heart rate of 122 bpm; other observations were within normal limits. Her abdomen was soft with generalized tenderness but no guarding or peritonism. A computed tomography (CT) scan of the abdomen and pelvis demonstrated diffuse mural thickening of the stomach and increased distension of the proximal small bowel, however no obstruction, collection or specific cause for the patient’s symptoms. She was admitted to the acute surgical unit for further investigation.

**Figure 1:**
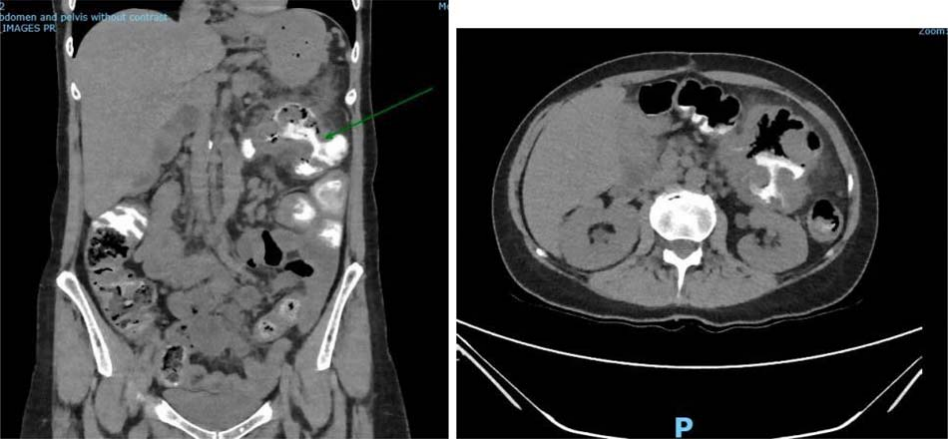
Coronal and axial slices of a CT abdomen/pelvis with oral contrast demonstrating a gastrocolonic fistula.

**Figure 2:**
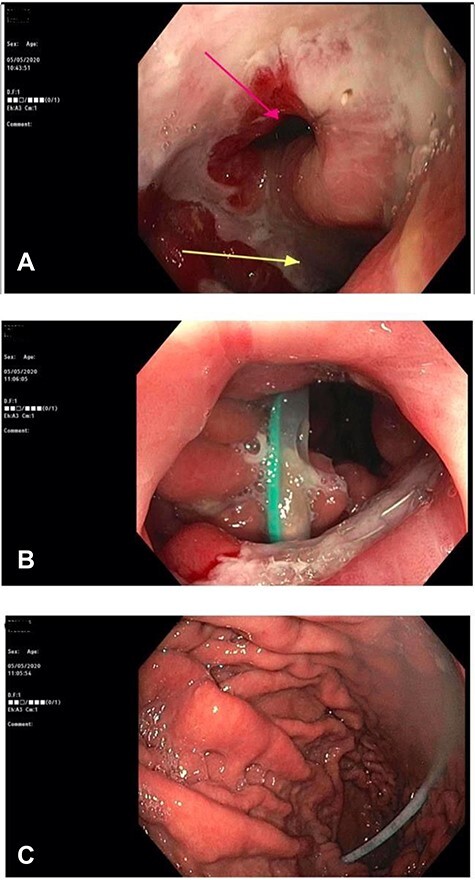
(**A**) Gastroscopy demonstrating a large gastrocolonic fistula (pink arrow) and gastrojejunostomy leading into the afferent jejunum (yellow arrow). (**B**) Image of colonoscopy at the splenic flexure demonstrating the gastrocolonic fistula with view of gastric mucosa and nasogastric tube. (**C**) The gastrocolonic fistula allowed passage of the colonoscope with relative ease. Image of gastric mucosa and nasogastric tube via the gastrocolonic fistula.

A repeat CT scan was performed, this time with oral contrast, which revealed a large gastrocolonic fistula into the transverse colon (Fig. 1). The patient underwent a gastroscopy and colonoscopy (Fig. 2A–C) which visually confirmed a 50 mm gastrocolonic fistula with a caliber sufficient to allow the passage of a colonoscope with relative ease (~13 mm), and no signs of active Crohn’s disease.

**Figure 3:**
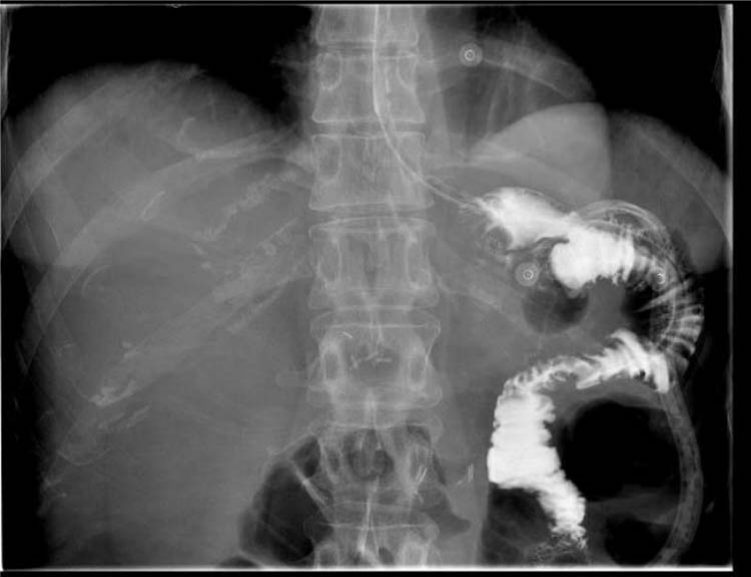
Gastrografin meal at 7 days post-operatively demonstrating resolution of gastrocolonic fistula.

Macroscopically there was no suggestion of malignancy or mass lesions; instead, it was felt that the fistula was likely caused by the advanced ulceration previously demonstrated on gastroscopy. The patient was commenced on total parenteral nutrition to optimize her nutrition preoperatively, and then 1 week after admission was taken to the operating theatre for definitive surgical management. At laparotomy, an inflammatory mass involving the distal stomach, transverse colon and Roux limb of the previous Roux-en-Y reconstruction was found. An uncomplicated near completion gastrectomy and segmental colectomy was performed, with the partially resected Roux limb able to be mobilized sufficiently to allow for the near-total gastrectomy to be appropriately reconstructed to the alimentary canal. Histopathology of the resected tissue was consistent with chronic inflammation and ulceration, with no evidence of malignancy. The patient recovered well post-operatively and a gastrograffin meal did not show any suggestion of ongoing fistulous communication or anastomotic leak (Fig. 3). She was discharged home Day 12 post-operatively on high-dose pantoprazole, and has since made an excellent recovery with resolution of her symptoms and improved nutritional status.

## DISCUSSION

Gastrocolonic fistulae form when a pathological communication develops between the stomach and colon, most commonly due to malignancy of either gastric or colonic origin. Benign causes include peptic ulcer disease, non-steroidal anti-inflammatory use, Crohn’s disease, chronic pancreatitis and, in rare cases, as a complication of upper gastrointestinal surgery [1]. To our knowledge, this is the first reported Australian case of a gastrocolonic fistula following upper gastrointestinal surgery. In this patient’s case, she underwent a pancreaticoduodenectomy for benign disease which was complicated by marginal ulceration, thought to be due to the effect of gastric acid on the exposed jejunal mucosa [2]. Despite aggressive medical and surgical management, the ulceration still progressed to a gastrocolonic fistula.

Classically gastrocolonic fistulae present with a triad of diarrhoea, feculent vomiting and weight loss, however this triad is only seen in ~30% of cases [3]. On presentation to ED, our patient reported all three symptoms described in the triad without radiological evidence of luminal obstruction. This is of note as the presentation of faeculent vomiting in the absence of obstruction should raise suspicion of a gastrocolonic fistula [4].

The limited literature on gastrocolonic fistulae suggests barium enemas are the most accurate imaging modality for diagnosis, with a close to 100% detection rate [1, 5]. Although the role of CT imaging has not been thoroughly explored in existing literature, CT with oral contrast successfully diagnosed this case with certain similarities existing between this and barium fluoroscopy modalities. By comparison, gastroscopy has a sensitivity of only 30–70% in terms of effective diagnosis, however, following the diagnosis of a fistula, endoscopy with biopsy is recommended to investigate for a malignant aetiology [3, 5].

Surgical resection of the fistula tract is the mainstay of treatment for gastrocolonic fistulae. A margin of adjacent tissue is typically taken to allow for both disease-free margins and a decreased risk of recurrence in the settings of malignant and benign disease respectively [1, 3]. In recent years, more experimental closure techniques have been reported, with varied success, including endoscopic repair with fistula luminal sinus clipping or fibrin glue [6–8]. Conservative management with suppression of gastric acid and nutrition optimization rarely leads to spontaneous closure. Nevertheless, success with this approach has been reported and may be preferred in patients who are unfit for surgery [8, 9].

## CONCLUSION

This is the first reported Australian case of a gastrocolonic fistula following upper gastrointestinal surgery. The rarity of these fistulae and non-specific presentation can make the diagnosis challenging, however the presence of feculent vomiting in the absence of intestinal obstruction should raise suspicion of a gastrocolonic fistula.
